# Weak Iron Oxidation by *Sulfobacillus thermosulfidooxidans* Maintains a Favorable Redox Potential for Chalcopyrite Bioleaching

**DOI:** 10.3389/fmicb.2018.03059

**Published:** 2018-12-12

**Authors:** Stephan Christel, Malte Herold, Sören Bellenberg, Antoine Buetti-Dinh, Mohamed El Hajjami, Igor V. Pivkin, Wolfgang Sand, Paul Wilmes, Ansgar Poetsch, Mario Vera, Mark Dopson

**Affiliations:** ^1^Centre for Ecology and Evolution in Microbial Model Systems, Linnaeus University, Kalmar, Sweden; ^2^Luxembourg Centre for Systems Biomedicine, University of Luxembourg, Belvaux, Luxembourg; ^3^Aquatic Biotechnology, Universität Duisburg-Essen, Essen, Germany; ^4^Faculty of Informatics, Institute of Computational Science, Università della Svizzera Italiana, Lugano, Switzerland; ^5^Swiss Institute of Bioinformatics, Lausanne, Switzerland; ^6^Plant Biochemistry, Ruhr-Universität Bochum, Bochum, Germany; ^7^College of Environmental Science and Engineering, Donghua University, Shanghai, China; ^8^Mining Academy and Technical University Freiberg, Freiberg, Germany; ^9^School of Biomedical and Healthcare Sciences, Plymouth University, Plymouth, United Kingdom; ^10^Schools of Engineering, Medicine and Biological Sciences, Institute for Biological and Medical Engineering, Pontificia Universidad Católica de Chile, Santiago, Chile; ^11^Department of Hydraulic and Environmental Engineering, School of Engineering, Pontificia Universidad Católica de Chile, Santiago, Chile

**Keywords:** redox control, microbial, bioleaching, chalcopyrite, iron oxidation, *Sulfobacillus*, *Leptospirillum*

## Abstract

Bioleaching is an emerging technology, describing the microbially assisted dissolution of sulfidic ores that provides a more environmentally friendly alternative to many traditional metal extraction methods, such as roasting or smelting. Industrial interest is steadily increasing and today, circa 15–20% of the world’s copper production can be traced back to this method. However, bioleaching of the world’s most abundant copper mineral chalcopyrite suffers from low dissolution rates, often attributed to passivating layers, which need to be overcome to use this technology to its full potential. To prevent these passivating layers from forming, leaching needs to occur at a low oxidation/reduction potential (ORP), but chemical redox control in bioleaching heaps is difficult and costly. As an alternative, selected weak iron-oxidizers could be employed that are incapable of scavenging exceedingly low concentrations of iron and therefore, raise the ORP just above the onset of bioleaching, but not high enough to allow for the occurrence of passivation. In this study, we report that microbial iron oxidation by *Sulfobacillus thermosulfidooxidans* meets these specifications. Chalcopyrite concentrate bioleaching experiments with *S. thermosulfidooxidans* as the sole iron oxidizer exhibited significantly lower redox potentials and higher release of copper compared to communities containing the strong iron oxidizer *Leptospirillum ferriphilum*. Transcriptomic response to single and co-culture of these two iron oxidizers was studied and revealed a greatly decreased number of mRNA transcripts ascribed to iron oxidation in *S. thermosulfidooxidans* when cultured in the presence of *L. ferriphilum*. This allowed for the identification of genes potentially responsible for *S. thermosulfidooxidans*’ weaker iron oxidation to be studied in the future, as well as underlined the need for new mechanisms to control the microbial population in bioleaching heaps.

## Introduction

Biomining is a sustainable process for metal extraction from sulfidic ores that has been studied by researchers around the globe since its emergence in the early 1950s (Temple and Colmer, 1951; Bryner and Jameson, 1958). In the recent decades and with its industrial application in mind, understanding of this natural process has significantly improved (Rohwerder et al., 2003; Watling, 2006; Vera et al., 2013; Jerez, 2017) and today, biomining is defined to be the microbial promoted oxidation of insoluble metal sulfides to acid soluble sulfates. In a technique termed bioleaching, this is undertaken to solubilize and recover metals of interest that form part of the metal sulfide mineral matrix. Ferrous iron (Fe^2+^)-oxidizing acidophilic microorganisms are responsible for the regeneration of the chemical oxidant ferric iron (Fe^3+^), which in turn attacks the sulfidic mineral, and breaks its covalent bonds. This releases ferrous iron plus any other contained metals and completes the catalytic cycle (Vera et al., 2013). While having initial economic disadvantages, mainly attributed to the long lag phase after construction of a bioleaching heap, biomining technologies are commonly considered more environmentally friendly than most conventional methods (Johnson, 2014). Today, increasing amounts of metals are extracted or processed by biomining technologies in many countries that include Chile, Australia, and South Africa, with the bioleaching of secondary copper sulfides accounting for an estimated 15–20% of the world wide copper production (Brierley and Brierley, 2013).

Bioleaching of primary copper minerals, such as the world’s most abundant copper mineral chalcopyrite (CuFeS_2_), remains challenging and suffers from slow dissolution rates. This is often attributed to the formation of passivation layers on the mineral surface (Cordoba et al., 2009; Wang et al., 2016), but it has also been argued that the semiconductor properties of chalcopyrite itself could be responsible (Crundwell, 2015). To date, extensive efforts to elucidate the exact nature of chalcopyrite passivation have not been successful (Khoshkhoo et al., 2014a, 2017). Despite this, strategies have been discovered to diminish the passivating effect, including bioleaching at high temperatures and low redox potentials (Li et al., 2013; Panda et al., 2015). Due to their large concentrations, the oxidation/reduction potential (ORP) in bioleaching systems is predominantly determined by the Fe^3+^/Fe^2+^ redox couple, whereby high concentrations of Fe^3+^ indicate high potentials. At low redox potentials and in the presence of millimolar concentrations of Fe^2+^ and Cu^2+^, chalcopyrite is suggested to be transformed into the secondary copper sulfide chalcocite (Cu_2_S) which is more readily oxidized by the Fe^3+^ provided by microbial action (Hiroyoshi et al., 2013). Methods to control the redox potential of the leaching solution include the addition of chemical reductants (Zhao et al., 2017) or limitation of oxygen (Third et al., 2002; Spolaore et al., 2009; Gericke et al., 2010). However, the technical realization of such methods in a large industrial bioheap with gradients of, e.g., temperature, oxygen concentration, and substrates has not been accomplished. Many studies have investigated the optimal microbial consortia in bioleaching operations (Rawlings and Johnson, 2007), usually focusing on the need to efficiently oxidize Fe^2+^ that drives the redox potential above that optimal for chalcopyrite dissolution (Hiroyoshi et al., 2013). In contrast, little attention has been paid to the possibility of controlling the redox potential of a bioleaching system by influencing the ratio of ferric to ferrous iron via suitable iron-oxidizing microbes (Masaki et al., 2018).

A large range of acidophile microbes have the capability to oxidize Fe^2+^ to gain energy under acidic conditions (Hedrich et al., 2011) and are therefore applicable in biomining operations. Among those are members of the *Acidithiobacillus*, *Acidimicrobium*, *Acidiferrobacter*, *Sulfobacillus*, and *Ferroplasma* genera (reviewed in Quatrini and Johnson, 2016). In bioleaching systems, one of the most abundant iron oxidizers is the moderately thermophilic, chemolithoautotroph *Leptospirillum ferriphilum* (Penev and Karamanev, 2010; Christel et al., 2017). This moderate thermophile solely derives its energy from the oxidation of ferrous iron (Coram and Rawlings, 2002) and is capable of doing so at very low Fe^2+^ ion concentrations and redox potentials as high as 700 mV vs. Ag/AgCl (Rawlings et al., 1999), giving it a significant advantage over other species. Another iron-oxidizer commonly found in acidic, sulfur rich environments is the moderately thermophilic *Sulfobacillus thermosulfidooxidans* (Karavaiko et al., 2005) that in contrast to *L. ferriphilum*, is unable to scavenge exceedingly scarce ferrous iron and is therefore considered a “weak” iron oxidizer in this study. In addition to Fe^2+^, *S. thermosulfidooxidans* is capable of oxidizing inorganic sulfur compounds (ISCs) and can utilize organic molecules to meet its carbon demands (Tsaplina et al., 2000). ISC oxidation is an important process in bioleaching heaps to remove excess sulfur compounds (Dopson and Lindstrom, 1999) and generate the necessary acidity, which is otherwise consumed by gangue minerals in low grade ores (Baldi et al., 1991; Dopson et al., 2009). Often, this role is fulfilled by obligate ISC-oxidizing species, such as the mesophile *Acidithiobacillus thiooxidans* or moderately thermophile *A. caldus* (Hallberg and Lindstrom, 1994).

In this study, we hypothesized that by inoculation of chalcopyrite ore with suitable iron-oxidizing bacteria the redox potential of the leachate in the initial phase of bioleaching experiments can be controlled. By these means, the redox potential can be maintained close to the optimum range. The initial rate of chalcopyrite dissolution is enhanced and thereby increases the amount of released copper. The applicability of this approach to industrial bioleaching operations is discussed.

## Materials and Methods

### Mineral

Chalcopyrite was provided by Boliden AB (Sweden) and originates from the Aitik copper mine (N 67° 4′ 24″, E 20° 57′ 51″), where traces of bornite (Cu_5_FeS_4_) are frequently found accompanying this mineral. The flotation concentrate used in this study was revealed to be of high purity (>98%) by *aqua regia* digestion and elemental analysis and contained 29.5% copper (Supplementary File 1). For bioleaching experiments, the concentrate was sieved to obtain the size fraction between 50 and 100 μm, and subsequently washed in three volumes of 0.1 M EDTA in 0.4 M NaOH for 10 min under stirring to remove iron and copper compounds originating from weathering of the mineral. Elemental sulfur was then removed from the surfaces by three iterations of washing with one volume of acetone. Finally, the mineral was dried at 60°C overnight and then sterilized at 120°C for 10 h under a nitrogen atmosphere to ensure no changes in the mineral structure occurred.

### Bacterial Strains and Growth Conditions

Three bacterial acidophile species were used in this study, *L. ferriphilum* DSM 14647^T^, *S. thermosulfidooxidans* DSM 9293^T^, and *A. caldus* DSM 8584^T^. Prior to the bioleaching experiments, cells were maintained in three separate axenic continuous cultures so that the cells were under the same growth state when all experiments were inoculated. The continuous cultures were maintained at 38°C, fed with MAC medium (Mackintosh, 1978), and electron donor added in the form of 100 mM ferrous sulfate (*L. ferriphilum*) or 5 mM potassium tetrathionate (*S. thermosulfidooxidans* and *A. caldus*). The continuous culture vessels, all tubing, plus MAC medium were autoclaved while the ferrous sulfate and potassium tetrathionate were sterile filtered (0.2 μm pore size, cellulose acetate filter, PALL).

### Bioleaching Experiments

Bioleaching experiments were conducted in quadruplets in 250 mL Erlenmeyer flasks. 100 mL MAC medium (adjusted to pH 1.8 by addition of sulfuric acid) was supplemented with 2% (wt/vol) chalcopyrite concentrate and inoculated with combinations of the three bacterial species (10^7^ cells per mL per species), obtained by centrifugation from the continuous cultures (12,500 ×*g*, 20 min) followed by cell counting using a Neubauer Improved counting chamber. Combinations included three single, three binary, and one tertiary combination, plus one sterile control. Cultures were incubated at 38 ± 2°C under slow shaking (120 rpm). Experiments were terminated 14 days after the first onset of microbial oxidation of ferrous iron as indicated by a redox potential >400 mV vs. Ag/AgCl, resulting in total incubation times ranging from 14 to 20 days.

Experiments were analyzed for pH (pHenomenal^®^ 221, VWR), redox potential (Ag/AgCl with 3 M KCl; InLab^®^ Redox-L, Mettler-Toledo), ferrous iron, total dissolved sulfur, elemental sulfur, as well as total iron and copper concentration in the leach liquor. Ferrous iron concentration was assessed by titration of its 1,10-phenanthroline complex (Walden et al., 1933; Dopson and Lindstrom, 1999). In short, 200 μL of bioleaching sample was centrifuged for 5 min at 16,000 ×*g*. Supernatant was mixed with the same volume of 15 mM 1,10-phenanthroline in aqueous 5 mM FeSO_4_, added to 1 mL of 1 M H_2_SO_4_ and subsequently titrated from orange to blue with 1 mM CeSO_4_. Total soluble and elemental sulfur were measured by photospectrometric measurement of thiocyanate complexes obtained by cyanolysis (Kelly et al., 1969) from the supernatant and pellet of a bioleaching sample, respectively. For total soluble sulfur, 500 μL sample was centrifuged for 5 min at 16,000 ×*g* and the supernatant mixed with 100 μL 0.5 M NaCN. After 10 min of incubation at room temperature, 500 μL of phosphate buffer (pH 7.2) and 100 μl 50 mM CuSO_4_ was added, followed by 30 min of incubation at room temperature. Then, complexes were formed by addition of 400 μL 1.5 M FeNO_3_ in aqueous 4 M HClO_4_ and distilled H_2_O to a volume of 2 mL. The reaction was then measured at 460 nm against a calibration curve of thiocyanate treated in the same way. For measurement of elemental sulfur, the mineral pellet of the same sample was dissolved in 2 mL of absolute acetone. 200 μL of this solution was then processed as described in the total soluble sulfur analysis, except that no CuSO_4_ was added, and the calibration curve was prepared using elemental sulfur dissolved in acetone. Total released, i.e., dissolved plus precipitated iron and copper concentrations were obtained by adding 1.8 mL of 5 M HCl to 200 μL of bioleaching slurry, followed by incubation at 65°C for 30 min. Then, the supernatant of this digest was diluted in 0.1 M HCl appropriate for measurement by atomic absorption spectroscopy (AAS) using a Perkin Elmer AAnalyst 400.

### Extraction and Analysis of Nucleic Acids

After 14 days of active bioleaching time (defined by a redox potential above 400 mV vs. Ag/AgCl), experiments were sampled for nucleic acid extraction. The flasks were left to settle for 5 min before removing 75 mL supernatant to be immediately mixed with an equal volume of sterile, ice-cold MAC medium. Then, the sample was centrifuged at 12,500 ×*g* for 20 min at 4°C. The resulting cell pellet was washed twice by resuspending in 10 and 2 mL of sterile, ice-cold MAC, respectively, and then frozen in liquid nitrogen. Cell pellets were subjected to biomolecular extractions according to a previously published method (Roume et al., 2013), skipping the metabolite extraction step. In short, cell pellets were lysed by cryo-milling and bead-beating followed by spin column based isolation of biomolecules with the Allprep kit (Qiagen, Belgium). Ribosomal RNA was depleted with the Ribo-Zero rRNA Removal Kit for bacteria (Illumina, United States). rRNA-depleted RNA for nine samples was stored at -80°C before shipping on dry ice to Science for Life Laboratory (Stockholm, Sweden) for sequencing.

### Sequence Analysis

Library preparation was performed with the Illumina TruSeq Stranded mRNA kit. Paired-end sequencing was performed on two HiSeq 2500 lanes resulting in on average 94 million reads per sample with length of 126 bp and GC% of 54% (Supplementary Table 1). Raw reads were filtered with Trimmomatic v0.32 (Bolger et al., 2014), TrueSeq3-PE adapter sequences were removed using the following parameters: seed mismatch:2; palindrome clip:30; simple clip:10; leading:20; trailing:20; sliding window: 1:3; minlen: 40; maxinfo: 40:0.5. Filtered reads were mapped onto a concatenation of the three reference genomes (*A. caldus* DSM 8584: GCF_000175575.2; *S. thermosulfidooxidans* DSM 9293: GCF_900176145.1; *L. ferriphilum* DSM 14647: GCF_900198525.1) with Bowtie-2 v2.3.2 (Langmead and Salzberg, 2012) with default parameters. Reads mapping to protein coding sequences were counted with the FeatureCounts program of the subread package v1.5.1 (Liao et al., 2014) with the –s 2 parameter accounting for strandedness. Read counts were then normalized and compared per organism with a custom R-script using the DESeq2 package v1.16.1 (Love et al., 2014) in R v3.4.4. Normalization was adapted from scripts provided in a previous publication (Klingenberg and Meinicke, 2017).

### Data Availability

Raw sequencing reads are available from ENA SRA under study accession PRJEB27534. Scripts used in the analysis of the sequencing data can be accessed under the following link: https://git-r3lab.uni.lu/malte.herold/RNAseq_LF_ST_redox. Lists of genes relevant for analysis were generated by manual curation of the reference genome annotations and from previous publications (Janosch et al., 2015; Christel et al., 2017).

## Results and Discussion

### Bioleaching of Chalcopyrite Concentrate

**FIGURE 1:**
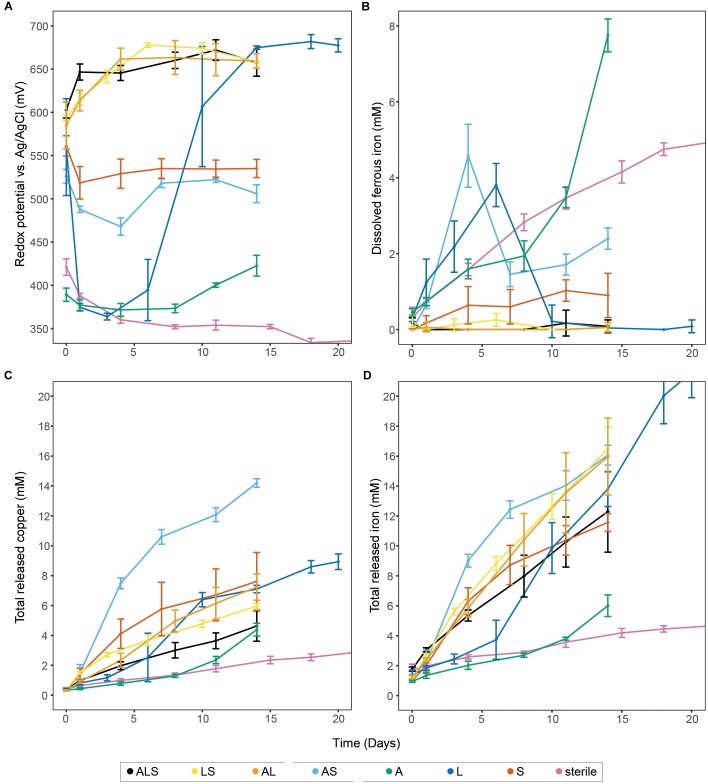
Bioleaching of chalcopyrite concentrate with single species, binary, and tertiary combinations of the three studied model species, plus uninoculated control. The panels show redox potential **(A)**, dissolved Fe^2+^ **(B)**, and total released copper and iron (**C,D**, respectively). Data points represent means ± SD (*n* = 4). Abbreviations in the legend denote: A, *A. caldus*, L; *L. ferriphilum*; and S, *S. thermosulfidooxidans*.

Bioleaching of chalcopyrite was tested with single, binary, and tertiary combinations of the three model species (*A. caldus*, *L. ferriphilum*, and *S. thermosulfidooxidans*), plus uninoculated controls to investigate the effect of species composition on redox potential and copper release (Figure 1 and Supplementary Figure 1). To aid comprehension, these combinations will be abbreviated using the initial letter of the included species (e.g., ‘ASL’ for the tertiary combination containing all species or ‘LS’ for the binary combination of *L. ferriphilum* and *S. thermosulfidooxidans*, etc.).

Physical and chemical analysis (Figure 1 and Supplementary Figure 1) of the uninoculated controls showed a redox potential of circa 310–330 mV (vs. Ag/AgCl) after stabilization, while the Fe^2+^ concentration steadily increased until plateauing at 5.7 ± 0.2 mM in later stages of the experiment (i.e., day 32, late stage data not shown). The abiotic leaching released a small amount of metal (4.2 ± 0.3 mM Fe and 2.4 ± 0.3 mM Cu after 15 days) from the chalcopyrite by proton attack and/or a small concentration of Fe^3+^ present on the mineral or in the medium. The same behavior was observed in the experiment inoculated exclusively with *A. caldus*, where the redox potential remained at circa 370 mV (i.e., well below the 400 mV perceived to mark the onset of bioleaching) until day 12 when likely environmental bacteria that survived the sterilization process on the mineral became active and commenced iron oxidation. Metal release was only marginally higher than from uninoculated controls, but showed a slight acceleration after the redox increase and reached 6.0 ± 0.7 mM Fe and 4.4 ± 0.4 mM Cu after 14 days. As sulfur compounds released from the mineral matrix are debated to be involved in formation of passivating layers (Khoshkhoo et al., 2014a,b), it is of importance to note that experiments containing, e.g., *A. caldus* exhibited examples of ISC degradation (Supplementary Figure 1), which is suggested to aid chalcopyrite dissolution (Spolaore et al., 2011). However, in the timeframe investigated during the leaching experiments, significant accumulation of soluble ISCs and elemental sulfur was also not observed in the ‘L’ combination excluding the sulfur oxidizers or in the sterile controls (Supplementary Figure 1). Beside the removal of passivating layers, ISC oxidation has the important role of maintaining low pH conditions in bioleaching heaps (Dopson and Lindstrom, 1999). Lacking sulfur oxidation potential in the microbial community can lead to a continuous rise in pH as observed in the sterile controls, or a potentially detrimental pH peak before the precipitation of ferric hydroxides and sulfates increases net acidity again (Ma et al., 2018; Supplemental Figure 1). This has implications for both the biology and engineering of bioleaching heaps, as many biomining species are sensitive to high pH, and accumulation of precipitates affect retention time of the leachate (Fagan et al., 2014). Additionally, pH induced precipitation of ferric iron alters the leachates ORP further affecting mineral dissolution.

Experiments including inoculation with iron-oxidizing bacteria allowed for transformation of Fe^2+^ to Fe^3+^ and therefore the redox potential rose up to, e.g., 682 ± 8 mV in the case of ‘L’. Accordingly, iron and copper release from all such experiments was significantly higher than from uninoculated controls and ‘A’; e.g., reaching the highest Fe concentration of 21.6 ± 1.7 mM in ‘L’ and highest copper concentration of 14.2 ± 0.3 mM in ‘AS’ (Figure 1).

**FIGURE 2:**
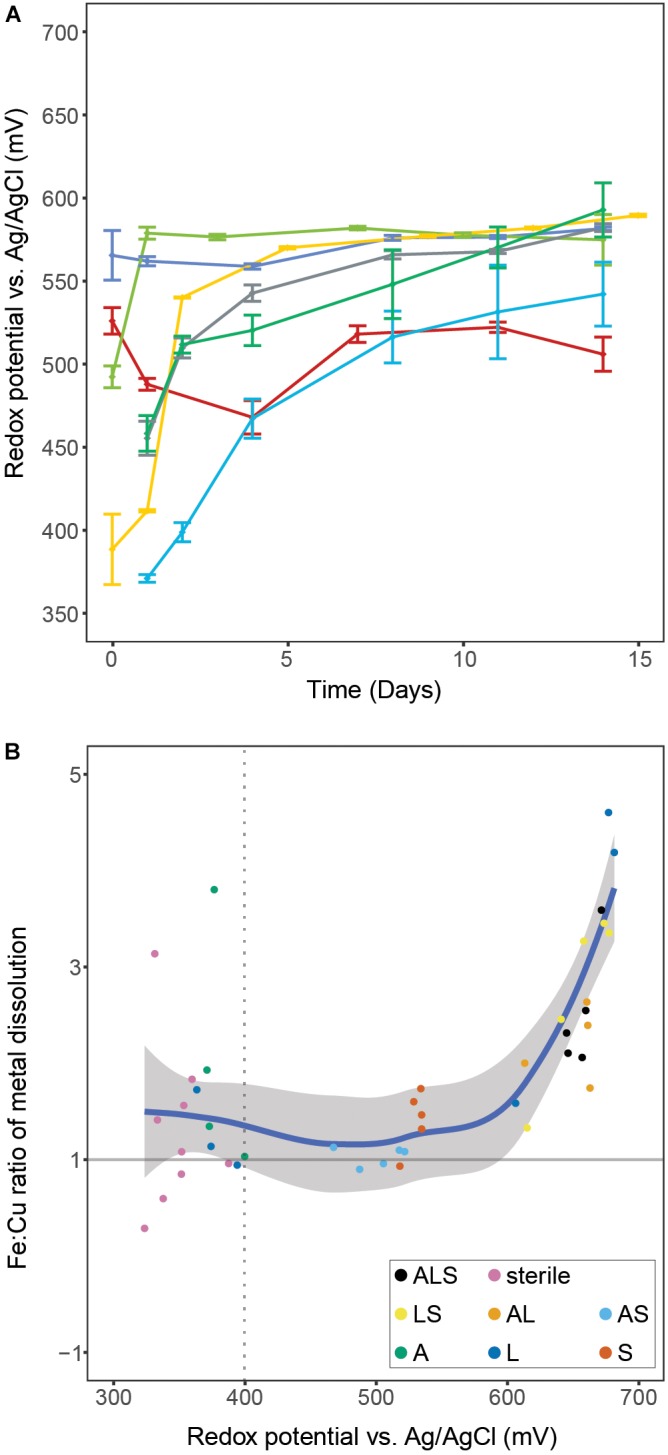
Panel **A** shows redox potentials remaining below 600 mV during seven independent experiments containing only *S. thermosulfidooxidans* for microbial iron oxidation. Data points represent means ± SD (*n* = 4). Panel **B** illustrates the correlation of the ratio of released iron:copper vs. redox potential during bioleaching of chalcopyrite concentrate with various combinations of the three model species. The ratio was calculated by dividing the amounts of the two metals that were released between two consecutive sampling points during the leaching experiments. The regression was calculated using the LOESS method with 95% confidence interval marked by the shaded area. The dotted line denotes the onset of microbial iron oxidation indicated by a redox potential above 400 mV. Abbreviations in the legend denote: A, *A. caldus*; L, *L. ferriphilum*; and S, *S. thermosulfidooxidans*.

The tertiary combination ‘ALS’ was outperformed by all binary combinations (Figure 1). This indicated that, in contrast to the currently accepted paradigm of inoculation of bioleaching applications with a broad mixture of biomining organisms, a well-chosen and defined mixture of microorganisms could benefit leaching efforts in the early stages of a bioleaching heap. Furthermore, the different combinations showed very distinct ORP profiles that, based on the present iron oxidizer(s), fell into one of two groups. All combinations containing *L. ferriphilum* had redox potentials between 650 and 680 mV compared to combinations in which it was excluded (i.e., ‘AS’ and ‘S’, showing ORPs below 550 mV). To confirm the lower redox potential in bioleaching cultures without *L. ferriphilum*, the ‘AS’ combination was repeated seven times with the redox potential reaching a maximum of 593 ± 15 mV (Figure 2A). Previous studies report that low redox potentials are favorable for chalcopyrite bioleaching (Third et al., 2002; Hiroyoshi et al., 2013). In accordance with that, in this study the redox potential of the leaching experiments correlated positively with the ratio of released iron/copper (Figure 2B). As dissolution of pure chalcopyrite is theoretically characterized by a 1:1 ratio of released iron to copper, this confirms the preferential oxidation of this copper mineral, or the transiently produced chalcocite, over associated copper-deficient minerals, such as pyrite, at low redox potentials. Our data independently confirms a study by Masaki et al. (2018), in which microbial redox control was also attempted, likewise by members of the Sulfobacilli, i.e., *S. sibiricus* and *S. acidophilus*. Using iron-oxidizing bacteria in bioleaching processes, which raise the ORP only minimally over the threshold for the onset of leaching therefore appears possible and could benefit the performance of chalcopyrite bioleaching processes.

Multiple reasons for the induction of diverse redox potentials by different species are conceivable. First and foremost, the effect could be explained by the effectivity and/or affinity of the respective species’ iron oxidation system. Species with a low affinity to ferrous iron, or inferior capability to oxidize it, should in theory maintain lower redox potentials. Additionally, high concentrations of Fe^3+^ ions are known to inhibit iron oxidation differently in different species (Rawlings et al., 1999), which could also contribute to this effect. Fluorescence microscopy examination of chalcopyrite grains in our bioleaching experiments revealed another difference that could contribute to the observed effect. *L. ferriphilum* showed significantly higher rates of colonization of mineral grains compared to *S. thermosulfidooxidans* (Bellenberg et al., 2018; Supplementary Figure 2). Attachment to metal sulfides is considered important for bioleaching, since in the so called ‘contact mechanism’, mineral-attached microbes concentrate Fe^3+^ in their EPS, effectively locally increasing the ORP at the microbe-mineral interface compared to the rest of the medium. Low levels of cell attachment on chalcopyrite mineral grains support the idea that a non-contact mechanism is observed for *S. thermosulfidooxidans*. Consequently, ferric ions diluted in the bulk medium maintain a more homogeneous redox environment. Unfortunately, Masaki et al. (2018) did not report on attachment rates and this hypothesis remains to be tested.

Additional factors affecting the iron oxidation efficiency could be the chosen experimental conditions. All used strains were adapted to grow at a temperature of 38°C prior to the bioleaching experiments, initially chosen as it is low enough to serve as an estimation of the temperature in a starting bioleaching heap (Leahy et al., 2007). At the same time, it is close to the optimum for *L. ferriphilum* that has a much narrower temperature range for growth while allowing reasonable growth for the other two species. Testing of *S. thermosulfidooxidans* iron oxidation efficiency near its growth optimum may become necessary, however, a raise in temperature has previously been reported to reduce leachate ORP in stirred reactors, and was connected to the exclusion of *L. ferriphilum* and retention of *S. thermosulfidooxidans* at a temperature of 48°C (Hedrich et al., 2018). Despite temperature control not being feasible in bioleaching heaps, this indicates that the low redox potential induced by *S. thermosulfidooxidans* in our experiments is not solely an effect of its growth at temperatures lower than its optimum. Overall, in the background of the other strain specific differences in experiments that run for prolonged periods of time, we consider the temperature influence as negligible, as the microbe’s growth capabilities are limited in the first place by the solubility of the mineral concentrates rather than temperature. This is strongly related to the acid-solubility of the mineral and to the iron oxidation capabilities of the respective single and mixed cultures. This is shown by all the iron being in the ferric form when leaching is efficient, i.e., the cells are limited by the availability of ferrous iron. Similar considerations were made for other parameters such as pH and carbon dioxide concentrations.

Owing to its heterogeneity and complexity, control of both chemical and biological parameters in a bioleaching heap is challenging (Petersen, 2016). Biologically, a major cause of this challenge is that due to implied costs, the mineral cannot be sterilized, and environmental bacteria will be present and thrive in the heap, competing with the inoculated ‘strategic’ microorganisms (Martinez et al., 2015). Procedures will have to be developed in order to fully exploit the potential of selected microorganisms, e.g., by continuously inoculating the heap with these strategic organisms through the irrigation system. In future studies, efforts should therefore be made to identify species with both low Fe^2+^ scavenging capabilities and increasingly high optimal growth temperatures.

In any case, manipulation of leachate, mineral, or other components of a bioleaching heap will naturally increase running costs. Further studies and ultimately large scale testing are needed to validate the viability of such approaches.

### Transcriptomic Analysis

**FIGURE 3:**
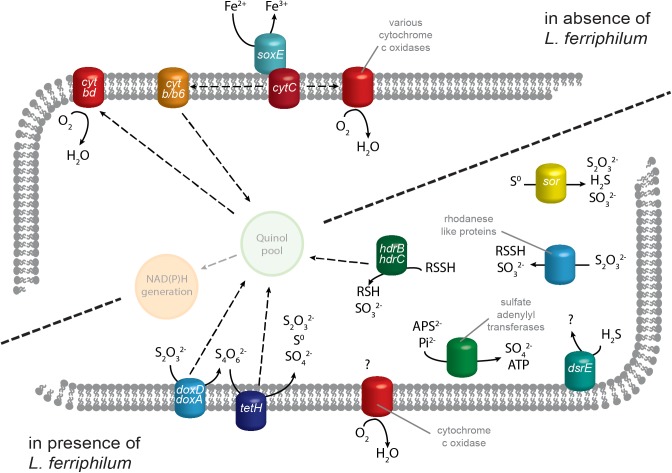
Proposed model of *S. thermosulfidooxidans* transcript regulation of genes related to energy generation. Iron oxidation systems and electron transport by cytochromes has a greater number of RNA transcripts in the absence of the strong iron oxidizer *L. ferriphilum*. In its presence, *S. thermosulfidooxidans* instead has higher transcript numbers for genes contributing to ISC oxidation plus one cytochrome *c* oxidase complex. Quinone pool and NAD(P)H generation are depicted translucently for comprehension, but corresponding genes were not analyzed in this study. Solid arrows represent metabolic reactions while dashed arrows indicate the relocation of electrons.

In an attempt to elucidate the biological background for the difference in redox potential, both iron-oxidizing model species’ transcriptomic response toward each other was investigated (i.e., ‘ASL’ vs. ‘AL’ for effect of *S. thermosulfidooxidans* on *L. ferriphilum*, and ‘ASL’ vs. ‘AS’ for the vice versa effect; Figure 3). The entirety of ecological interactions between the involved species is beyond the scope of this study and instead, this section concentrates on gene products related to energy metabolism, iron-, and sulfur oxidation as previously reported (Mangold et al., 2011; Guo et al., 2014; Christel et al., 2017; Supplementary Tables 1, 2).

#### *L. ferriphilum* and *A. caldus*

In bioleaching co-culture, *L. ferriphilum* remained remarkably unaffected by the presence of *S. thermosulfidooxidans*. Over its entire genome (2486 genes), only 36 genes showed significant differential expression in response to *S. thermosulfidooxidans* (‘AL’ vs. ‘ALS’; *p* ≤ 0.05; data not shown). Among the 26 genes attributed to iron oxidation and electron transport, merely three *cbb*_3_-type cytochrome *c* oxidase subunits (LFTS_01396, _02094, and _02276) exhibited significantly increased transcript numbers in the presence of *S. thermosulfidooxidans*, all of which have log2-fold changes below 1.5 (Supplementary Table 2). No genes involved in iron oxidation or electron transport had significantly higher numbers of RNA transcripts in the absence of *S. thermosulfidooxidans*.

*Acidithiobacillus caldus* exhibited 508 significantly differentially expressed genes (of 2885) depending on the iron oxidizer present (‘AL’ vs. ‘AS’; *p* ≤ 0.05; data not shown). Of these, 246 showed increased transcript counts during co-culture with *S. thermosulfidooxidans*. Significant changes related to energy acquisition included a strong increase in relative RNA transcript counts for a large portion of the sulfur oxidizing *soxABXYZ* and likely associated cytochrome *c* biogenesis protein genes (Supplementary Table 3). This was surprising as, although *S. thermosulfidooxidans* is capable of sulfur oxidation, due to its preference for iron oxidation in the absence of stronger iron oxidizers (see following sections), this species should not be in direct competition with *A. caldus* in this case. This may potentially be explained if *A. caldus* maintains a high level of sulfur oxidation proteins in the presence of other potential sulfur oxidizing microbes so as not to be outcompeted. In contrast, the presence of *L. ferriphilum* appeared to enhance transcription of cytochrome *bd* ubiquinol oxidase subunits I and II (Supplementary Table 3). In addition to its respiratory function, cytochrome *bd* complex has been reported to increase resistance against oxidative stress in *Escherichia coli* (Lindqvist et al., 2000) and could fulfill a similar function in the high ORP conditions induced by *L. ferriphilum*.

#### *S. thermosulfidooxidans* Fe^2+^ Oxidation and Electron Transport

*Sulfobacillus thermosulfidooxidans* gene transcript numbers exhibited great variation depending on presence of *L. ferriphilum*. Of its 3805 identified genes, 828 showed significant differential expression (‘AS’ vs. ‘ALS’; *p* ≤ 0.05; data not shown). Among the 83 selected genes involved in iron oxidation, electron transport, and sulfur oxidation, 55 had significantly greater or lower RNA transcripts (Table 1 and Supplementary Table 4). Large variation was observed in genes related to iron oxidation. In contrast to, e.g., some members of the genus *Acidithiobacillus*, Sulfobacilli genomes lack the common iron oxidation protein rusticyanin (Guo et al., 2014). Instead, Sulfobacilli are suggested to utilize sulfocyanin, which is also found in the archaeal iron oxidizers of the genus *Ferroplasma* (Dopson et al., 2005). In the presence of *L. ferriphilum*, *S. thermosulfidooxidans* strongly decreased transcript numbers attributed to two of the five *soxE* genes coding for this protein (Sulth_0453 and _2749). Additionally, the vast majority of identified cytochromes of all types exhibited decreased transcript counts, along with corresponding biogenesis proteins and quinol oxidases (Table 1 and Supplementary Table 4). The strong downregulation of electron chain components that were likely linked to iron oxidation in *S. thermosulfidooxidans* could be explained by the chemical data reported in the previous section. In cultures containing both iron oxidizers, the concentration of available ferrous iron was below the detection limit and likely too low for utilization by *S. thermosulfidooxidans*. This may be attributed to *L. ferriphilum* being able to scavenge Fe^2+^ at concentrations far below *S. thermosulfidooxidans*’ capabilities and at large Fe^3+^ concentrations that exceed its inhibition limits (Rawlings et al., 1999).

**Table 1:** Excerpt of Supplementary Table 1 showing significant (|log2FC| ≥ 1.0, *p* ≤ 0.05) differential expression of *S. thermosulfidooxidans* genes related to iron and sulfur oxidation as well as electron transport.

Gene ID	Product	DESeq normalized expression				log2FC
		AS mean	AS STD	ASL mean	ASL STD	
**Iron oxidation and electron transport chain**						
Sulth_0051	Cytochrome *c* assembly protein	1086	91	2412	150	−1.15
Sulth_0119	Cytochrome *c* class I	250	72	105	37	1.25
Sulth_0449	Heme/copper-type cytochrome/quinol oxidase, subunit 3	5850	537	919	85	2.67
Sulth_0450	Cytochrome *c* oxidase subunit I	15675	2453	2857	266	2.46
Sulth_0451	Cytochrome *c* oxidase subunit II	15243	1526	4700	545	1.70
Sulth_0453	Sulfocyanin (SoxE)	7722	884	623	112	3.63
Sulth_0488	Cytochrome *c* oxidase subunit I	17287	3212	533	106	5.02
Sulth_0489	Cytochrome *c* oxidase subunit II	12771	1543	405	58	4.98
Sulth_0494	Cytochrome *d* ubiquinol oxidase, subunit II	161	11	57	38	1.50
Sulth_0495	Cytochrome *bd* ubiquinol oxidase subunit I	355	102	39	10	3.17
Sulth_0840	Cytochrome *c* oxidase, *cbb*_3_-type, subunit III	557	173	81	30	2.78
Sulth_0843	Heme/copper-type cytochrome/quinol oxidase, subunit 3	154	20	24	6	2.67
Sulth_0844	Cytochrome *c* oxidase subunit I	431	29	35	14	3.62
Sulth_0845	Cytochrome *c* oxidase subunit II	228	4	30	6	2.93
Sulth_1456	Cytochrome *c* oxidase subunit II, periplasmic domain	86	11	43	8	1.01
Sulth_1490	Cytochrome *c* oxidase, cbb3-type, subunit III	76	22	22	6	1.79
Sulth_1513	Cytochrome *c* oxidase subunit II	15255	1578	4733	457	1.69
Sulth_1514	Cytochrome *c* oxidase subunit I	34803	3976	16822	1585	1.05
Sulth_1901	Cytochrome *c* biogenesis protein	442	46	999	136	−1.18
Sulth_1930	Cytochrome *c* oxidase subunit IV	408	92	4081	275	−3.32
Sulth_1931	Cytochrome *c* oxidase subunit III	507	64	4862	339	−3.26
Sulth_1932	Cytochrome *c* oxidase subunit I	1427	269	15277	462	−3.42
Sulth_1933	Cytochrome *c* oxidase subunit II	1764	452	17994	1718	−3.35
Sulth_2044	Cytochrome *c* class I	91	31	18	9	2.36
Sulth_2183	Cytochrome *c* biogenesis protein transmembrane region	291	108	816	311	−1.49
Sulth_2568	Cytochrome *c*-type biogenesis protein CcmE	123	36	47	3	1.37
Sulth_2572	Cytochrome *c*-type biogenesis protein CcmB	68	22	14	3	2.28
Sulth_2573	Cytochrome *c* assembly protein	114	15	12	7	3.33
Sulth_2730	Cytochrome *b*/*b*_6_ domain	819	19	238	110	1.78
Sulth_2731	Cytochrome *b*/*b*_6_ domain protein	2148	652	804	64	1.42
Sulth_2749	Sulfocyanin (SoxE)	9756	2642	1112	151	3.13
**Sulfur metabolism**						
Sulth_0921	Pyrrolo-quinoline quinone repeat-containing protein, tetH	613	101	25972	7210	−5.41
Sulth_0946	FAD-dependent pyridine nucleotide-disulfide oxidoreductase, Sqr_1	207	45	75	11	1.46
Sulth_1024	Hypothetical protein	125	50	57	8	1.13
Sulth_1025	Heterodisulfide reductase, subunit C, *hdrC*	24	0	50	8	−1.08
Sulth_1433	Sulfate adenylyltransferase	369	88	1430	384	−1.95
Sulth_1435	Sulfate adenylyltransferase	252	31	1202	289	−2.26
Sulth_1627	Sulfur oxygenase/reductase, Sor	591	29	1340	319	−1.18
Sulth_1878	Rhodanese-like protein	176	36	381	49	−1.12
Sulth_2076	Rhodanese-like protein	203	23	416	37	−1.03
Sulth_2172	Rhodanese-like protein	3024	1076	12511	2067	−2.05
Sulth_2770	Heterodisulfide reductase, subunit C, *hdrC*	11592	2924	29882	1777	−1.37
Sulth_2771	Heterodisulfide reductase, subunit B, *hdrB*	13422	4935	28681	4989	−1.10
Sulth_2782	DsrE family protein	4123	1767	14140	4441	−1.78
Sulth_3251	Pyrrolo-quinoline quinone repeat-containing protein, *tetH*	163	5	1551	223	−3.25

*Negative log2-fold changes indicate higher transcript in presence of L. ferriphilum (ASL), positive changes upregulation in its absence (AS). Mean expression values are calculated from three independent experiments (n = 3). Abbreviations: STD, standard deviation; log2FC, log2-fold change.*

Contrary to this overall trend, one gene cluster of *S. thermosulfidooxidans* encoding cytochrome *c* oxidase subunits I-IV showed strongly increased transcript counts in the presence of *L. ferriphilum* (Table 1, Sulth_1930-1933). In addition, two cytochrome *c* biogenesis proteins (Sulth_1901 and _2183) and one cytochrome *c* assembly protein (Sulth_0051) exhibited similarly increased transcript numbers. A direct role of cytochromes in iron oxidation has been suggested in an acid mine drainage biofilm and in *L. ferrooxidans* (Jeans et al., 2008; Blake and Griff, 2012). Therefore, the strong opposite regulation of cytochrome oxidases in *S. thermosulfidooxidans* raises the question of their potential functional and/or structural differences. It could be possible that the oxidase exhibiting increased transcript counts in the presence of *L. ferriphilum* indirectly facilitates a higher affinity for Fe^2+^, or has a lower sensitivity toward oxidative stress induced by accumulating Fe^3+^. Together with the upregulation of biogenesis and assembly proteins, this could enable *S. thermosulfidooxidans* to gain some energy from Fe^2+^ in the presence of a stronger iron oxidizer. Alternatively, the cytochrome *c* oxidase complex upregulated in the presence of *L. ferriphilum* could be part of the strong upregulation of genes described in the following section, i.e., reducing oxygen in the final step of sulfur oxidation systems. Nevertheless, as only cytochromes and cytochrome oxidases that are upregulated in absence of *L. ferriphilum* correlated with higher copper extraction, they may be of greater interest in the context of this study and should be considered in more detail in the future.

#### *S. thermosulfidooxidans* ISC Oxidation

Genes coding for known sulfur oxidation proteins exhibited directionally opposite changes in transcript numbers compared to iron oxidation systems. Conceivably, this was to ensure sufficient supply of energy in a Fe^2+^ deficient environment and the vast majority of *S. thermosulfidooxidans* genes related to sulfur metabolism (Guo et al., 2014) had significantly higher RNA transcripts in the presence of *L. ferriphilum* (Table 1 and Supplementary Table 4). This reiterates that in presence of a strong iron oxidizer, *S. thermosulfidooxidans*’ role lies in the removal of ISCs as discussed by Spolaore et al. (2011). The highest of these log2-fold changes were recorded for two copies of tetrathionate hydrolase gene *tetH* (Sulth_0921 and _3251) while a third copy (Sulth_1188) exhibited moderately increased transcript counts in the absence of *L. ferriphilum*. TetH is responsible for the hydrolysis of tetrathionate, an important intermediate in sulfide mineral dissolution. Additionally, thiosulfate can be oxidized by thiosulfate quinone oxidoreductase, encoded by a *doxDA* homolog. The two encoded copies of this gene exhibited increased transcript counts when in co-culture with *L. ferriphilum*, although the log2-fold changes were low (Sulth_1989 and 1691). A similar function is suggested for rhodanese-like proteins, which in humans are sulfur transferases involved in the detoxification of cyanide by transformation to thiocyanate in the liver (Nakajima, 2015). Proteins sharing their active domain are also suggested to play a role in microbial sulfur oxidation, in particular of thiosulfate (Valdes et al., 2008). In *S. thermosulfidooxidans*, seven genes coding for such proteins were present (Table 1 and Supplementary Table 4) and four of them exhibited significantly increased transcript numbers in the presence of *L. ferriphilum* (Sulth_1878, _2076, _2172, and _3294). The product of their enzymatic reaction can be further oxidized by heterodisulfide reductase, which is encoded in *S. thermosulfidooxidans* by three sets of genes for its respective subunits HdrBC (Supplementary Table 4). Four Hdr subunit gene loci exhibited significant slightly increased transcript counts in presence of *L. ferriphilum* (Sulth_1025, _1026, _2770, and _2771). Elemental sulfur oxidation is also relevant in the context of sulfide mineral dissolution, and is conducted by the product of two copies of sulfur oxygenase/reductase gene *sor* (Janosch et al., 2015). While one copy was only minimally expressed in both conditions (Sulth_1798; indicating a possibly defunct gene), the second exhibited greater transcript numbers (Sulth_1627) and appeared to be enhanced by the presence of *L. ferriphilum*.

Additional contributions to sulfur oxidation systems include sulfate adenylyltransferase, which is suggested to be involved in sulfite oxidation in *A. ferrooxidans*^T^ and *S. thermosulfidooxidans* strain ST (Guo et al., 2014) and is likely to fulfill the same role in *S. thermosulfidooxidans*^T^. Similarly, DsrE-family protein (Sulth_2782) has been reported to be associated to oxidative sulfite metabolism (Dahl et al., 2005) and was also found to exhibit increased transcript numbers in presence of *L. ferriphilum* (Table 1).

In the absence of *L. ferriphilum*, only a few genes related to sulfur oxidation were significantly enhanced. Of note in this regard is one of three present copies of sulfide quinone reductase gene *sqr1* (Sulth_0946), which is responsible for the oxidation of sulfide to elemental sulfur a hypothetical protein within the *hdr* gene cluster (Sulth_1024), as well as the upper mentioned copy of *tetH* (Sulth_1188).

## Conclusion

During bioleaching of chalcopyrite concentrate, *S. thermosulfi-dooxidans* but not *L. ferriphilum* maintained a low redox potential that is favorable for the extraction of copper. We hypothesize that this was due to differences in affinity and/or effectivity of the species’ respective iron oxidation systems, as well as the attachment rate of the microorganisms to the mineral grains. This finding could potentially contribute to overcoming passivation and improving dissolution rates in large-scale chalcopyrite bioleaching. Expression of iron and sulfur oxidation systems in *S. thermosulfidooxidans* were investigated during bioleaching experiments in presence and absence of *L. ferriphilum*. Presence of the strong iron oxidizer induced greatly decreased transcript counts attributed to iron oxidation and increased counts for sulfur oxidation. Analysis of this data revealed genes products potentially responsible for the difference in ORP, which should be studied in this regard in the future. Additionally, this study underlines the importance of developing methods to control microbial populations in a bioleaching heap in order to exploit desired properties of selected microorganisms.

## Author Contributions

SC conducted the laboratory experiments and drafted the manuscript. MH performed bioinformatic analysis of transcript data. SB provided microscopic imaging. SC, SB, AB-D, MEH, IP, WS, PW, AP, MV, and MD were involved in data analysis and biological interpretation of the results. All authors contributed to its preparation.

## Conflict of Interest Statement

The authors SC, MH, SB, AB-D, MEH, IP, PW, AP, and MD and/or the institutions in which they are affiliated with, have a patent pending based on the results of the study. The remaining authors declare that the research was conducted in the absence of any commercial or financial relationships that could be construed as a potential conflict of interest.
