# Supplementary material for: Weak Iron Oxidation by Sulfobacillus thermosulfidooxidans Maintains a Favorable Redox Potential for Chalcopyrite Bioleaching
Source: Front Microbiol. 2018 Dec 12;9:3059. doi: 10.3389/fmicb.2018.03059 (PMC6315122; doi:10.3389/fmicb.2018.03059)
Supplement: Supplementary file 1 [file Data_Sheet_1.PDF]

Supplementary Material

Weak Iron Oxidation by Sulfobacillus thermosulfidooxidans Maintains a Favorable Redox Potential for Chalcopyrite Bioleaching

Stephan Christel[1*], Malte Herold[2], Sören Bellenberg[3], Antoine Buetti-Dinh[4,5], Mohamed El Hajjami[6], Igor V. Pivkin[4,5], Wolfgang Sand[3,7,8] , Paul Wilmes[2], Ansgar Poetsch[6,9], Mario Vera[10] and Mark Dopson[1]

*** Correspondence:** Stephan Christel, stephan.christel@lnu.se

Supplementary Table 1 Overview of sequencing results. Nine samples were sequenced, species composition can be seen in the “Sample_Name” in the first of five fields separated by “-“. A = *A. caldus*, L = *L. ferriphilum*, S = *S. thermosulfidooxidans*, X = no third species. Read numbers or numbers of read pairs are given in million (M). Read length for raw reads was 126bp and GC% 54 % on average.

Sample ID	Sample Name	Raw read pairs	Filt. read pairs	Single-tons after filtering	Filt. reads total	Reads passing filtering	Reads map-ped	Filt. reads map-ped	Reads assigned to CDS	Mapped reads assigned to CDS
		M	M	M	M	%	M	%	M	%
P9658_101	ALX9-Si00-14A-P-R1	53.7	41.6	11.8	95.0	88.45	79.3	83.47	31.9	40.23
P9658_102	ALX9-Si00-14C-P-R1	41.5	31.3	10.0	72.6	87.47	58.8	80.99	22.1	37.59
P9658_103	ALX9-Si00-14D-P-R1	40.0	29.0	10.8	68.8	86.00	65.8	95.64	26.1	39.67
P9658_104	ASL9-Si00-14B-P-B-R1	44.1	34.1	9.7	77.9	88.32	65.5	84.08	24.8	37.86
P9658_105	ASL9-Si00-14C-P-B-R1	36.4	30.4	5.8	66.6	91.48	58.7	88.14	18.4	31.35
P9658_106	ASL-Si00-14D-P-R1	40.2	32.2	7.9	72.3	89.93	62.2	86.03	24.6	39.55
P9658_107	ASX9-Si00-14A-P-B-R1	40.2	30.8	9.2	70.8	88.06	60.2	85.03	22.0	36.54
P9658_108	ASX9-Si00-14B-P-B-R1	41.1	32.2	8.7	73.1	88.93	62.3	85.23	23.7	38.04
P9658_109	ASX9-Si00-14C-P-B-R1	71.4	38.2	32.0	108.4	75.91	69.8	64.39	25.2	36.10

Supplementary Table 2 Differential expression of *L. ferriphilum* genes related to iron oxidation and electron transport. Negative log_2-fold changes indicate higher transcript numbers in presence of *S. thermosulfidooxidans* (ASL), positive changes upregulation in its absence (AL). Mean expression values are calculated from three independent experiments (n = 3). Abbreviations: std, standard deviation; log_2FC, log_2 fold change.

Gene ID	Product	Deseq normalized expression				log2FC	p
		AL mean	AL std	ASL mean	ASL std		
**Iron oxidation and electron transport chain**							
LFTS_00439	Cytochrome d ubiquinol oxidase subunit I	142	14	318	100	-1.17	1.00
LFTS_00440	Cytochrome d ubiquinol oxidase subunit II	62	10	129	8	-1.06	NA
LFTS_00441	cyd operon protein YbgT	24	5	45	4	-0.92	NA
LFTS_00730	Cytochrome c	777	130	568	37	777	1.00
LFTS_00731	Cytochrome c	532	85	463	34	532	NA
LFTS_00732	Cytochrome c553	378	46	312	29	378	1.00
LFTS_00740	Cytochrome c553	716	90	696	17	0.04	NA
LFTS_00741	Cytochrome c	787	36	959	54	-0.28	1.00
LFTS_00766	Cytochrome c mono- and diheme variants	350	73	395	31	-0.18	NA
LFTS_00821	Menaquinol-cytochrome c reductase iron-sulfur subunit	374	54	534	40	374	NA
LFTS_00822	Ubiquinol-cytochrome c reductase cytochrome b subunit	561	45	771	53	561	NA
LFTS_00940	Cytochrome c554 and c-prime	1027	196	1114	27	-0.12	1.00
LFTS_01351	Cytochrome c oxidase cbb3-type subunit III	324	17	249	26	0.38	1.00
LFTS_01388	Cytochrome c	743	225	941	95	-0.34	1.00
LFTS_01396	Cytochrome c oxidase cbb3-type subunit 1	3503	1058	7479	613	-1.09	0.01
LFTS_01531	Cytochrome c oxidase cbb3-type subunit 2	20581	2376	27877	1619	-0.44	0.09
LFTS_01857	Cytochrome c oxidase cbb3-type subunit 1	42303	2216	58664	2231	-0.47	0.44
LFTS_01930	Cytochrome c oxidase cbb3-type subunit III	226	40	289	50	-0.35	NA
LFTS_01943	Cytochrome b6-f complex iron-sulfur subunit	4162	549	4159	529	0.00	1.00
LFTS_01964	Cytochrome c oxidase cbb3-type subunit 1	1040	185	768	60	0.44	1.00
LFTS_01972	Cytochrome c oxidase cbb3-type subunit 2	8874	466	12947	881	-0.54	0.70
LFTS_02094	Cytochrome c oxidase cbb3-type subunit III	14344	3749	29365	1968	-1.03	<0.01
LFTS_02276	Cytochrome c oxidase cbb3-type subunit III	10429	641	20234	3857	-0.96	0.01
LFTS_02378	Cytochrome c-type biogenesis protein	526	161	777	116	-0.56	1.00
LFTS_02381	Cytochrome c-type biogenesis protein CcsB	2175	110	1783	52	0.29	1.00
LFTS_02382	Cytochrome c biogenesis protein	5071	279	4138	516	0.29	1.00

Supplementary Table 3 Differential expression of *A. caldus* genes related to sulfur oxidation and electron transport. Negative log_2-fold changes indicate higher transcript numbers in presence of *S.thermosulfidooxidans* (AS), positive changes upregulation in presence of *L. ferriphilum* (AL). Mean expression values are calculated from three independent experiments ($n = 3$). Abbreviations: std, standard deviation; log_2FC, log_2 fold change.

Gene ID	Product	Deseq normalized expression				log2FC	p
		AS mean	AS std	AL mean	AL std		
**Sulfur metabolism**							
cds0279	Cysteine synthase	418	195	438	86	0.07	0.93
cds0940	TetR family transcriptional regulator	1704	320	4960	657	1.54	<0.01
cds1169	Sulfur oxidation protein	214	54	770	145	1.85	<0.01
cds1314	Tetrathionate hydrolase	1162	425	1598	239	0.46	0.21
cds1450	Sulfurtransferase	4124	465	3336	310	-0.31	0.11
cds1673	DsrE family protein	603	58	1165	217	0.95	<0.01
cds2017	Sulfur oxidation c-type cytochrome SoxA	12222	3470	1728	610	-2.82	<0.01
cds2018	Sulfur oxidation c-type cytochrome SoxX	4886	855	975	320	-2.33	<0.01
cds2021	Thiosulfohydrolase SoxB	55847	8600	5414	1547	-3.37	<0.01
cds2022	Thiosulfate oxidation carrier complex protein SoxZ	21825	3294	1853	527	-3.56	<0.01
cds2023	Thiosulfate oxidation carrier protein SoxY	27841	4145	2690	812	-3.37	<0.01
cds2148	Disulfide reductase	13899	2662	10915	212	-0.35	0.15
cds2149	Heterodisulfide reductase subunit C	9224	1997	8792	424	-0.07	0.86
cds2152	Heterodisulfide reductase subunit B	24607	3598	20673	902	-0.25	0.23
cds2153	Heterodisulfide reductase subunit C	22032	1921	20282	857	-0.12	0.66
cds2156	Sulfurtransferase	304	50	459	115	0.6	0.04
cds2166	Sulfur oxidation c-type cytochrome SoxX	1561	359	130	37	-3.59	<0.01
cds2167	Thiosulfate oxidation carrier protein SoxY	1311	105	127	37	-3.36	<0.01
cds2168	Thiosulfate oxidation carrier complex protein SoxZ	1832	304	152	51	-3.59	<0.01
cds2169	Sulfur oxidation c-type cytochrome SoxA	2482	531	301	54	-3.04	<0.01
cds2171	Thiosulfohydrolase SoxB	3228	725	459	16	-2.82	<0.01
cds2195	Heterodisulfide reductase subunit B	22796	5923	16930	2034	-0.43	0.13
cds2404	TetR family transcriptional regulator	2287	907	2140	508	-0.1	0.87
**Electron transport**							
cds0049	Cytochrome c biogenesis protein	6736	1143	4460	498	-0.59	<0.01
cds0471	Cytochrome d ubiquinol oxidase subunit II	708	177	1976	309	1.48	<0.01
cds0472	Cytochrome d ubiquinol oxidase subunit I	1398	778	5625	390	2.01	<0.01
cds0662	Cytochrome o ubiquinol oxidase	21945	2069	12305	1709	-0.83	<0.01
cds0663	Cytochrome o ubiquinol oxidase	30999	2384	16707	1514	-0.89	<0.01
cds0664	Cytochrome o ubiquinol oxidase	6004	606	3319	504	-0.85	<0.01
cds0666	Cytochrome c oxidase assembly protein	5665	673	3071	514	-0.88	<0.01
cds0728	Cytochrome d ubiquinol oxidase subunit II	1041	734	5554	1023	2.42	<0.01
cds0729	Cytochrome d ubiquinol oxidase subunit I	1802	1227	10966	2176	2.61	<0.01
cds1300	Cytochrome d ubiquinol oxidase subunit II	77	21	128	29	0.74	0.42
cds1301	Cytochrome d ubiquinol oxidase subunit I	231	41	319	54	0.46	0.74
cds1321	Cytochrome d ubiquinol oxidase subunit I	2030	315	4418	1136	1.12	<0.01
cds1322	Cytochrome d ubiquinol oxidase subunit II	1315	224	2137	481	0.7	0.01
cds1348	Cytochrome c	12438	2323	8381	2002	-0.57	0.02
cds1980	Cytochrome d ubiquinol oxidase subunit II	836	490	2406	279	1.53	<0.01
cds1981	Cytochrome d ubiquinol oxidase subunit I	1851	807	8004	961	2.11	<0.01
cds2016	Cytochrome c biogenesis protein	6131	985	1324	366	-2.21	<0.01
cds2019	Cytochrome c biogenesis protein ResB	12451	1751	1800	556	-2.79	<0.01
cds2026	Cytochrome o ubiquinol oxidase	4281	523	3870	990	-0.15	0.78

cds2027	Cytochrome o ubiquinol oxidase subunit I	19854	3126	16028	2147	-0.31	0.3
cds2028	Cytochrome o ubiquinol oxidase	18836	2612	20525	4325	0.12	0.8
cds2085	Cytochrome bd oxidase subunit I	6839	976	15351	7550	1.17	<0.01
cds2086	Cytochrome bd oxidase subunit II	2833	169	4858	1096	0.78	<0.01
cds2121	Cytochrome c biogenesis protein	11084	1998	1105	268	-3.33	<0.01
cds2504	Cytochrome c biogenesis protein	7902	2373	885	115	-3.16	<0.01
cds2505	Cytochrome c biogenesis protein	4515	1004	837	41	-2.43	<0.01
cds2865	cytochrome C oxidase assembly protein	517	24	636	204	0.3	0.38
cds2866	Cytochrome o ubiquinol oxidase	1682	751	2018	100	0.26	0.66
cds2867	Cytochrome o ubiquinol oxidase	1357	611	1940	229	0.52	0.27
cds2868	Cytochrome o ubiquinol oxidase	7099	3090	7743	576	0.13	0.85
cds2869	Cytochrome o ubiquinol oxidase	5079	1317	5876	239	0.21	0.71

Supplementary Table 4 Differential expression of *S. thermosulfidooxidans* genes related to iron oxidation and electron transport, as well as sulfur oxidation. Negative log_2-fold changes indicate higher transcript numbers in presence of *L. ferriphilum* (ASL), positive changes upregulation in its absence (AS). Mean expression values are calculated from three independent experiments ($n = 3$). Abbreviations: std, standard deviation; log_2FC, log_2 fold change.

**Gene ID**	**Product**	**Deseq normalized expression**				**log2FC**	**p**
		**AS mean**	**AS std**	**ASL mean**	**ASL std**		
**Iron oxidation and electron transport chain**							
Sulth_0051	Cytochrome c assembly protein	1086	91	2412	150	-1.15	<0.01
Sulth_0119	Cytochrome c class I	250	72	105	37	1.25	<0.01
Sulth_0443	Cytochrome c oxidase caa3-type, assembly factor CtaG-related	2717	432	2728	322	-0.01	0.98
Sulth_0449	Heme/copper-type cytochrome/quinol oxidase, subunit 3	5850	537	919	85	2.67	<0.01
Sulth_0450	Cytochrome c oxidase subunit I	15675	2453	2857	266	2.46	<0.01
Sulth_0451	Cytochrome c oxidase subunit II	15243	1526	4700	545	1.70	<0.01
Sulth_0453	Sulfocyanin (SoxE)	7722	884	623	112	3.63	<0.01
Sulth_0488	Cytochrome c oxidase subunit I	17287	3212	533	106	5.02	<0.01
Sulth_0489	Cytochrome c oxidase subunit II	12771	1543	405	58	4.98	<0.01
Sulth_0494	Cytochrome d ubiquinol oxidase, subunit II	161	11	57	38	1.50	<0.01
Sulth_0495	Cytochrome bd ubiquinol oxidase subunit I	355	102	39	10	3.17	<0.01
Sulth_0600	Cytochrome d ubiquinol oxidase, subunit II	1118	172	1032	68	0.12	0.56
Sulth_0601	Cytochrome bd ubiquinol oxidase subunit I	1801	250	2379	211	-0.40	0.03
Sulth_0840	Cytochrome c oxidase, cbb3-type, subunit III	557	173	81	30	2.78	<0.01
Sulth_0843	Heme/copper-type cytochrome/quinol oxidase, subunit 3	154	20	24	6	2.67	<0.01
Sulth_0844	Cytochrome c oxidase subunit I	431	29	35	14	3.62	<0.01
Sulth_0845	Cytochrome c oxidase subunit II	228	4	30	6	2.93	<0.01
Sulth_0932	Heme/copper-type cytochrome/quinol oxidase, subunit 3	33	5	35	8	-0.11	0.80
Sulth_0933	Cytochrome c oxidase subunit I	80	11	84	28	-0.09	0.80
Sulth_0934	Cytochrome c oxidase subunit II	16	5	8	4	0.94	0.42
Sulth_1456	Cytochrome c oxidase subunit II, periplasmic domain	86	11	43	8	1.01	<0.01
Sulth_1488	Sulfocyanin (SoxE)	1713	92	2057	214	-0.26	0.12
Sulth_1490	Cytochrome c oxidase, cbb3-type, subunit III	76	22	22	6	1.79	<0.01
Sulth_1511	Sulfocyanin (SoxE)	11643	1197	13389	1081	-0.20	0.16
Sulth_1513	Cytochrome c oxidase subunit II	15255	1578	4733	457	1.69	<0.01
Sulth_1514	Cytochrome c oxidase subunit I	34803	3976	16822	1585	1.05	<0.01
Sulth_1515	Heme/copper-type cytochrome/quinol oxidase, subunit 3	12061	190	8390	348	0.52	<0.01
Sulth_1801	Cytochrome-c3 hydrogenase	13178	682	10848	611	0.28	0.07
Sulth_1840	Cytochrome b/b6 domain	3145	337	2127	128	0.56	<0.01
Sulth_1853	Cytochrome bd ubiquinol oxidase subunit I	312	96	215	8	0.53	0.25
Sulth_1854	Cytochrome d ubiquinol oxidase, subunit II	344	135	227	30	0.60	0.13
Sulth_1901	Cytochrome c biogenesis protein	442	46	999	136	-1.18	<0.01
Sulth_1930	Cytochrome c oxidase subunit IV	408	92	4081	275	-3.32	<0.01
Sulth_1931	Cytochrome c oxidase subunit III	507	64	4862	339	-3.26	<0.01
Sulth_1932	Cytochrome c oxidase subunit I	1427	269	15277	462	-3.42	<0.01
Sulth_1933	Cytochrome c oxidase subunit II	1764	452	17994	1718	-3.35	<0.01
Sulth_2044	Cytochrome c class I	91	31	18	9	2.36	<0.01
Sulth_2121	Cytochrome c biogenesis protein transmembrane region	926	53	552	9	0.75	<0.01
Sulth_2183	Cytochrome c biogenesis protein transmembrane region	291	108	816	311	-1.49	<0.01
Sulth_2232	Sulfocyanin (SoxE)	568	46	618	44	-0.12	0.41
Sulth_2568	Cytochrome c-type biogenesis protein CcmE	123	36	47	3	1.37	<0.01
Sulth_2569	Cytochrome c assembly protein	340	67	224	49	0.61	0.01
Sulth_2572	Cytochrome c-type biogenesis protein CcmB	68	22	14	3	2.28	<0.01

Sulth_2573	Cytochrome c assembly protein	114	15	12	7	3.33	<0.01
Sulth_2706	Cytochrome oxidase assembly	3070	301	3396	53	-0.15	0.23
Sulth_2730	Cytochrome b/b6 domain	819	19	238	110	1.78	<0.01
Sulth_2731	Cytochrome b/b6 domain protein	2148	652	804	64	1.42	<0.01
Sulth_2746	Sulfocyanin (SoxE)	2119	1271	1178	279	0.85	0.22
Sulth_2749	Sulfocyanin (SoxE)	9756	2642	1112	151	3.13	<0.01
Sulth_2974	Cytochrome C oxidase, cbb3-type, subunit III	17	7	6	3	1.53	0.13
**Sulfur metabolism**							
Sulth_0548	FAD-dependent pyridine nucleotide-disulfide oxidoreductase, Sqr_1	4521	1132	5921	702	-0.39	0.17
Sulth_0580	FAD-dependent pyridine nucleotide-disulfide oxidoreductase, Sqr_1	2530	195	2281	273	0.15	0.34
Sulth_0921	Pyrrolo-quinoline quinone repeat-containing protein, tetH	613	101	25972	7210	-5.41	<0.01
Sulth_0946	FAD-dependent pyridine nucleotide-disulfide oxidoreductase, Sqr_1	207	45	75	11	1.46	<0.01
Sulth_1021	Heterodisulfide reductase, subunit C, hdrC	27	19	49	10	-0.87	0.09
Sulth_1022	Heterodisulfide reductase, subunit B, hdrB	58	19	84	19	-0.54	0.24
Sulth_1023	FAD-dependent pyridine nucleotide-disulphide oxidoreductase	49	12	71	25	-0.52	0.26
Sulth_1024	Hypothetical protein	125	50	57	8	1.13	0.01
Sulth_1025	Heterodisulfide reductase, subunit C, hdrC	24	0	50	8	-1.08	0.01
Sulth_1026	Heterodisulfide reductase, subunit B, hdrB	62	5	109	26	-0.82	0.03
Sulth_1046	DsrE family protein	182	95	273	18	-0.58	0.15
Sulth_1188	Pyrrolo-quinolinequinone repeat-containing protein, tetH	2808	306	1552	224	0.85	<0.01
Sulth_1366	Sulfate adenylyltransferase	482	23	629	27	-0.38	0.07
Sulth_1433	Sulfate adenylyltransferase	369	88	1430	384	-1.95	<0.01
Sulth_1435	Sulfate adenylyltransferase	252	31	1202	289	-2.26	<0.01
Sulth_1627	Sulfur oxygenase/reductase, Sor	591	29	1340	319	-1.18	<0.01
Sulth_1680	Rhodanese-like protein	255	40	159	23	0.68	<0.01
Sulth_1689	TQO small subunit DoxD domain-containing protein	181	54	225	8	-0.31	0.39
Sulth_1691	TQO small subunit DoxA domain-containing protein	288	64	522	115	-0.86	<0.01
Sulth_1798	Sulfur oxygenase/reductase, Sor	15	2	7	3	1.00	0.10
Sulth_1859	Sulphur transport	458	113	313	53	0.55	0.22
Sulth_1878	Rhodanese-like protein	176	36	381	49	-1.12	<0.01
Sulth_2076	Rhodanese-like protein	203	23	416	37	-1.03	<0.01
Sulth_2172	Rhodanese-like protein	3024	1076	12511	2067	-2.05	<0.01
Sulth_2335	Rhodanese-like protein	803	167	779	63	0.04	0.86
Sulth_2350	DsrE/DsrF-like family	364	22	300	2	0.28	0.16
Sulth_2674	DrsE family protein	1787	388	1347	236	0.41	0.06
Sulth_2770	Heterodisulfide reductase, subunit C, hdrC	11592	2924	29882	1777	-1.37	<0.01
Sulth_2771	Heterodisulfide reductase, subunit B, hdrB	13422	4935	28681	4989	-1.10	<0.01
Sulth_2782	DsrE family protein	4123	1767	14140	4441	-1.78	<0.01
Sulth_3040	Rhodanese-like protein	655	81	756	40	-0.21	0.19
Sulth_3251	Pyrrolo-quinolinequinone repeat-containing protein, tetH	163	5	1551	223	-3.25	<0.01
Sulth_3294	Rhodanese-like protein	273	41	351	45	-0.36	0.05

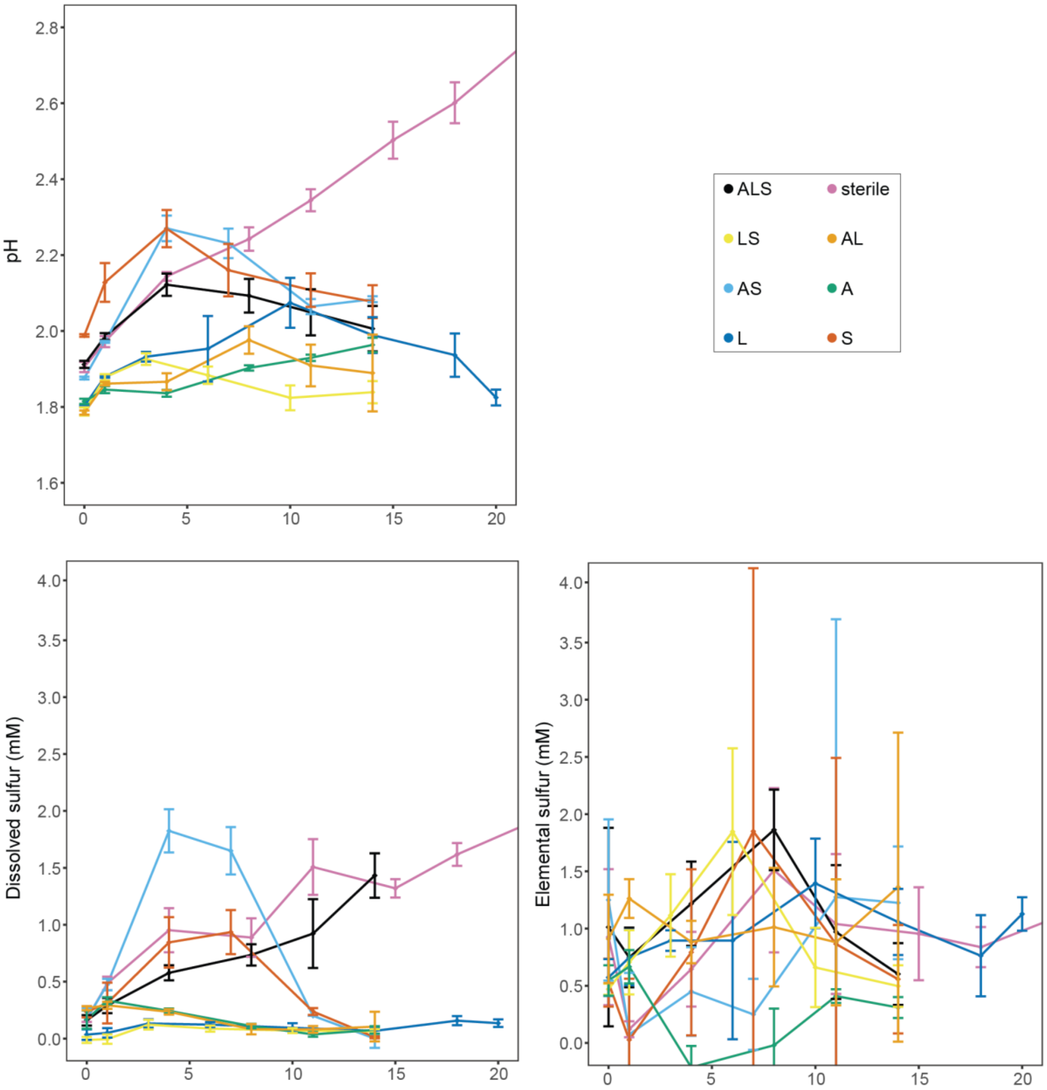

Supplementary Figure 1 Development of additional chemical parameters not reported in Figure 1, measured during bioleaching experiments using all seven species combinations, plus sterile control. Data points represent means ± standard deviations (n = 4). Abbreviations in the legend denote: A = *A. caldus*, L = *L. ferriphilum*, and S = *S. thermosulfidooxidans*.

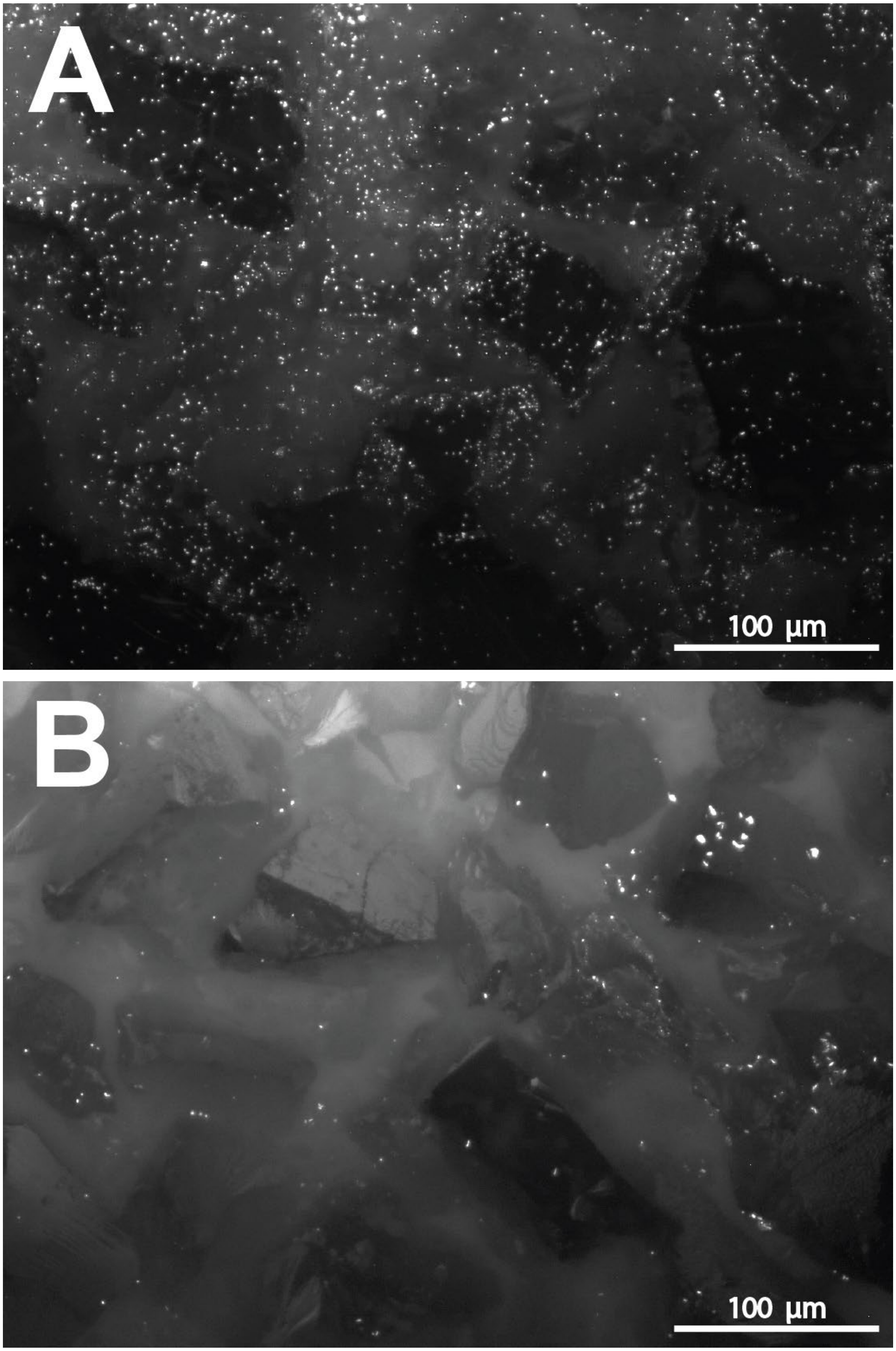

Supplementary Figure 2 Fluorescence microscopy image of cells attached to chalcopyrite grains after 12 days of inoculation with *L. ferriphilum* (A) or *S. thermosulfidooxidans* (B).
